# Experiences and satisfaction of children, young people and their parents with alternative mental health models to inpatient settings: a systematic review

**DOI:** 10.1007/s00787-019-01420-7

**Published:** 2019-10-21

**Authors:** Frane Vusio, Andrew Thompson, Max Birchwood, Latoya Clarke

**Affiliations:** Warwick Medical School, University of Warwick, Coventry, CV4 7AL UK

**Keywords:** Children and young people, Alternatives to inpatient settings, Mental health crisis, Experiences and satisfaction, Crisis intervention, Parental experiences

## Abstract

Community-based mental health services for children and young people (CYP) can offer alternatives to inpatient settings and treat CYP in less restrictive environments. However, there has been limited implementation of such alternative models, and their efficacy is still inconclusive. Notably, little is known of the experiences of CYP and their parents with these alternative models and their level of satisfaction with the care provided. Therefore, the main aim of this review was to understand those experiences of the accessibility of alternative models to inpatient care, as well as overall CYP/parental satisfaction. A searching strategy of peer-reviewed articles was conducted from January 1990 to December 2018, with updated searches conducted in June 2019. The initial search resulted in 495 articles, of which 19 were included in this review. A narrative synthesis grouped the studies according to emerging themes: alternative models, tele-psychiatry and interventions applied to crisis, and experiences and satisfaction with crisis provision. The identified articles highlighted increased satisfaction in CYP with alternative models in comparison with care as usual. However, the parental experiential data identified high levels of parental burden and a range of complex emotional reactions associated with engagement with crisis services. Furthermore, we identified a number of interventions, telepsychiatric and mobile solutions that may be effective when applied to urgent and emergency care for CYP experiencing a mental health crisis. Lastly, both parental and CYP experiences highlighted a number of perceived barriers associated with help-seeking from crisis services.

**Electronic supplementary material:**

The online version of this article (10.1007/s00787-019-01420-7) contains supplementary material, which is available to authorized users.

## Introduction

The latest 2017 survey of mental health prevalence for children and young people (CYP) in England showed that approximately 12.5% of 5–19 year olds were affected by adverse mental health issues [1]. In addition, despite the high prevalence of mental health disorders among CYP in the UK, help-seeking rates among CYP are in decline [2, 3]. Furthermore, there are evident treatment gaps, with up to 55% of adolescents aged 12–15 not receiving access to Children and Adolescent Mental Health Services (CAMHS) [4]. The treatment gap is similar for 16–20 year olds, whilst it may be as high as 64% for 21–25 year olds [4]. Moreover, a survey showed that 35% of young people (YP) requiring mental health services did not have any contact with them [5]; primarily due to insufficient resources within CAMHS services and a reluctance amongst some CYP to engage with CAMHS services [5].

Consequently, the high prevalence rates of mental health disorders amongst CYP aged 0–25 are applying significant pressures to inpatient settings and emergency departments struggling to cope with these increasing numbers [6, 7]. As result of this high demand for mental health provision, the quality of mental health services in emergency departments and inpatient settings is declining [2]. Moreover, a lack of beds in inpatient settings [7, 8] is resulting in increasing numbers of CYP being sent to adult inpatient services that are inappropriate for their needs [9]. Additionally, many CYP are also admitted to inpatient settings that are miles away from their places of residence, which can negatively impact their mental health outcomes and recovery [8]. All these factors may contribute towards a decline in CYP help-seeking behaviours and an increase in the number of CYP experiencing mental health crisis [10, 11].

To decrease the pressures on emergency departments and inpatient settings, there is a growing area of research that proposes the utilisation of alternative models for CYP in crisis that are capable to intervene early and prevent the escalation of mental health issues through less restrictive and community-based approaches [12–15]. These particular alternatives to inpatient settings could help develop more cost-effective services that could act as gatekeepers towards the admittance of CYP to inpatient settings [14, 16].

In 2008, a systematic review conducted by Shepard et al. [13] identified eight worldwide commonly used alternative models to inpatient care for CYP with complex mental health needs. These particular models were classified as multisystemic therapy, day hospitals, intensive specialist outpatient service (including crisis intervention and rapid outreach), home treatments, family preservation/wraparound services, case management, temporary residential care and therapeutic foster care provision [13, 17]. Despite a lack of high-quality evidence, Shepard’s review concluded that these models may be suitable alternatives to inpatient settings [13, 17].

Similar alternative models are well employed across the UK, such as intensive home treatments, early intervention services for psychosis, assertive outreach; intensive day services and outpatient treatment, day hospitals, therapeutic foster care and crisis intervention services [17]. Nevertheless, a review conducted in 2012 indicated that these alternative models vary widely in structure, with inconclusive methodological evidence rated as low or very low for their clinical effectiveness [15]. A similar conclusion came from another review, stating that “there is little systematic evidence of efficacy” of intensive community services (ICS) as an alternative to inpatient settings [12]. However, ICS may be considered a possible alternative approach with very limited evidence, which according to Kwok et al. [12] is focused predominantly on data generated from YP with moderate-to-severe levels of mental health needs.

From this literature review, it was visible that positive steps have been made towards the improvement of alternatives to inpatient settings and that there is an increasing focus on community-based services. However, the effectiveness of these alternatives still remains unclear. Nevertheless, there is some evidence that such alternatives and community-based models could be suitable substitutes to inpatient settings. However, to our best knowledge, no systematic review has explicitly examined the experiences and satisfaction of CYP and their parents during the time they were accessing urgent and emergency mental health services. Additionally, we are still not sure whether there are any newly developed models or interventions, since these reviews were published, that have more unique approaches towards prevention of hospitalisation or inpatient admission.

Therefore, this systematic review aims to focus on the following questions: (1) what are the experiences and satisfaction of CYP and their parents, with mental health crisis services or alternatives to inpatient settings? (2) What are the identified interventions that can be applied to CYP in urgent and emergency environments? (3) Besides well-established and known models, are there any newly developed alternative models to inpatient or emergency department admissions for CYP experiencing mental health crisis?

## Methods

This systematic review was both conducted and reported following the PRISMA guidelines [18]. The systematic review protocol for this review was submitted and approved by PROSPERO (ID: CRD42019110875).

## Search strategy

The present searching strategy was expanded upon from previously conducted systematic reviews [12, 13, 15]. We developed our search strategy based on terms relating to ‘*alternatives to inpatient settings*’, ‘*urgent and emergency mental health provision*’, ‘*children and young people*’, and ‘*patient satisfaction*’. The searching strategy (Table 1) was conducted on Embase, Medline and Psychinfo, Scopus; Web of Science; CINAHL and ASSIA databases.

**Table 1:** Example of searching strategy applied to Ovid Medline

	Search strategy
a)	((Child OR adolescen$ OR youth$ OR teenage$ OR ‘young people’)
AND	
b)	(mental health crisis OR mental health crises OR (mental health emergency OR mental health emergencies) OR (psychiatric adj (crisis OR crises OR emergenc* OR acute OR intensive)) OR (mental$ adj disorder$) OR (mental$ adj ill$) OR psychopathology)
AND	
c)	(ambulatory care OR residential treatment OR home care service$ OR psychiatric hospital* OR community mental health service* OR inpatient* OR community service* OR wraparound OR psychotherapy OR early intervention OR crisis intervention OR foster home care OR continuity of patient care OR (alternative adj(inpatient or in-patient)) OR assertive community treatment* OR mobile mental health crisis OR (multi-systemic or mulitsystemic) OR virtual mental health OR respite centre OR outpatient treatment OR child$ mental health service$ OR mental health treatment* OR mental health hospital admission OR mental health treatment outcome*)
AND	
d)	(user experience OR subjective experience OR patient satisfaction OR patient perspective))

The last rerun of the searching strategy was completed in June 2019 and resulted in no additional papers. Besides the searching strategy, we also conducted forward and backward manual searches applied to the studies that met the inclusion criteria. The backward searches helped us identify and examine references cited in the articles, while forward searching allowed us to identify any recent publications made by authors of studies that met inclusion criteria after publication of their article.

## Eligibility criteria

During the process of assessing the suitability of screened articles, the following inclusion criteria were applied: studies published between January 1st 1990 and December 20th 2018 predominantly on CYP who had experiences of acute mental health or mental health crisis. Additional criteria included parents or carers of CYP who experienced acute mental health; models and interventions that could be applied to both mental health crisis and alternatives to inpatient settings or could improve inpatient admission and reduce the length of stay.

Studies were included where at least 50% of the sample comprised of CYP aged 0–25. Studies were excluded if they involved patients older than 25 or reported on staff perceptions. Systematic reviews, book chapters, dissertations, grey literature, and articles on young offenders and learning disabilities, or those that were published in other languages than English were also excluded.

## Study selection

All articles taken from the seven electronic databases were transferred into the software ‘Rayyan’ [19], which was used for their analysis. Once all duplicates were removed, titles and abstracts were screened independently by two researchers (FV and LC). Any study that met the inclusion criteria was screened by full text, again independently by two researchers (FV and LC). Any disagreement between the researchers was handled by involving a third party (AT). The decision of the third party was considered final.

## Quality assessment and risk of bias

The quality of the included articles was assessed by the Mixed Methods Appraisal Tool [20]. The MMAT is a critical appraisal tool that is suitable for both qualitative, quantitative and mixed-method studies [20]. According to Hong et al. [20], the MMAT “permits to appraise the methodological quality of five categories to studies: qualitative research, randomised controlled trials, non-randomized studies, quantitative descriptive studies, and mixed methods studies”. Due to the lack of research evidence in this particular area, and as recommended by Hong et al. [20], we did not exclude studies with low methodological quality from this systematic review.

Appraised studies were classified into three categories according to their quality: low, medium and high. Studies were rated high if all five MMAT criteria were met. In the event that a study met four or three criteria, the study was classified as medium, i.e. meeting some criteria. Lastly, in the event that a study met one or two criteria, the study was classified as low quality, i.e. meeting minimum criteria. If any study did not meet the MMAT minimum screening criteria [20], the study was still included and reported, but without the MMAT screening result. We found two papers that did not pass MMAT minimum screening criteria [21, 22].

## Data extraction

Initially developed and piloted on a smaller sample of studies, the data extraction form was later adopted and used on the 19 identified articles. Our results are divided into four main themes, with the following data extraction information: authors, publishing year, country of origin, model or intervention name, study design, age and sample size, key findings, outcomes and satisfaction data. Two reviewers independently carried out data extraction (FV and LC).

## Data synthesis

We adopted a three-stage narrative synthesis approach as described by Popay et al. [23] in which the first stage starts with the development of the preliminary synthesis of findings of included studies. In the second stage, it is recommended to explore relationships both within and between studies, while the third stage requires an assessment of the robustness of the synthesis. As the studies covered by this systematic review had significant differences with their methodological approaches, a meta-analysis was not feasible. Nevertheless, the qualitative studies were analysed by re-occurring themes and subthemes.

## Results

### Study selection

**Fig. 1:**
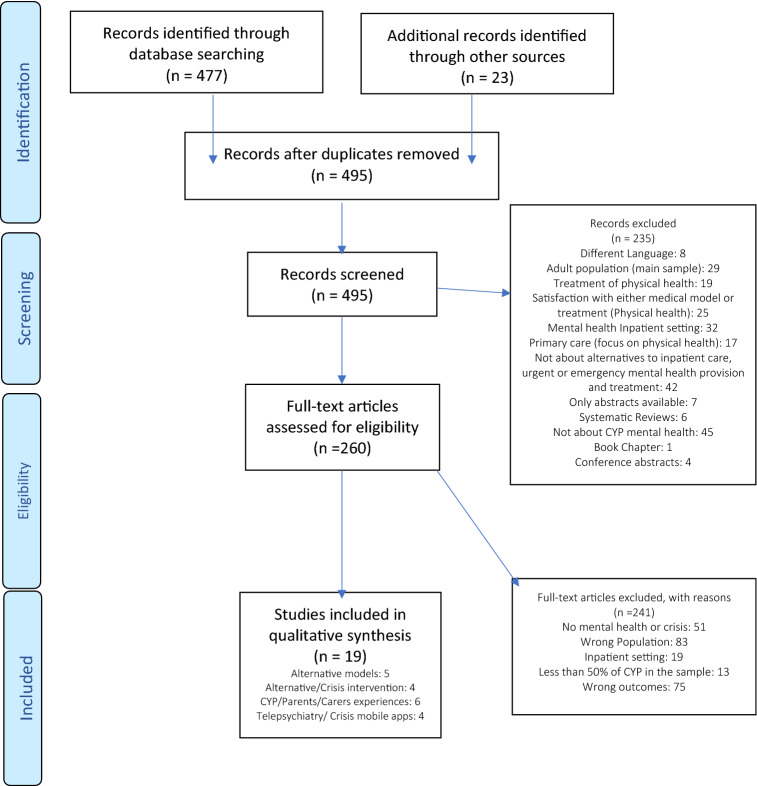
Prisma flowchart selection process

Our search strategy identified a total of 477 articles, from which an additional 23 articles were identified using both forward and backward manual searches of reference lists. Following the removal of duplicates, 260 articles were selected for full-text examination, while 235 articles were excluded. Common reasons for the exclusion of these articles were due to non-CYP study populations, a focus on inpatient settings, and a lack of relevance to CYP mental health, amongst others (Fig. 1). Of the 260 articles that were fully screened, 19 studies were independently chosen for inclusion by both reviewers. There were no disagreements. The full selection process is presented in the PRISMA flowchart [24] (Fig. 1).

### Study characteristics

The 19 studies included in this review came from 5 different countries; 8 studies came from the UK [21, 22, 25–30], 5 from the US [31–35], 3 from Australia [36–38], 2 from Canada [39, 40] and 1 from Denmark [41]. Eight studies utilised a qualitative methodology [25, 28, 29, 34, 36–38, 40] and two studies were based on a qualitative case-study approach [22, 33]. In contrast, three studies followed a quantitative descriptive approach [30, 35, 39], while one study had a mixed-methods design [26]. Two studies followed an RCT design [27, 31] and two studies were non-randomised with their methodological approach [21, 32]. Lastly, one study was identified as an RCT protocol [41]. Only three studies [25, 36, 37] included experiential data obtained from parents and relatives, while all others involved only CYP between 0 and 25. The sample size of CYP in the included papers ranged from 5 to 1397. Detailed information of the included studies are available in “Appendix”.

### Synthesis of results

The final sample comprised of 19 articles included in this review and provides outcome evidence in the following four domains: alternative models [21, 26, 27, 29, 30]; interventions applied to Crisis [22, 31–33]; telepsychiatry and mobile applications applied to mental health crisis [35, 38, 39, 41]; and experience and satisfaction with mental health crisis provision [25, 28, 34, 36, 37, 40].

#### Alternative models

We identified five alternative models based in the UK:

- *The York model* is a multidisciplinary, fully integrated community-based model that works in partnership with both statutory and voluntary sectors to provide multi-agency provision for CYP within the UK [26]. The main advantages of this model lie predominantly in its accessibility, responsiveness, single point of entry, 24/7 urgent and emergency provision for CYP, and fully integrated service which enables smooth navigation through care pathways for CYP [26]. These features of the model reduce the need for re-referrals, as all the services are closely integrated, which consequently prevents CYP to fall through the gaps between the services [26].
- A similar multi-agency approach was taken by *the UK One Stop Shop model*, a nurse-led drop-in clinic for CYP who are affected by ADHD [30]. Even though similar ‘one stop shop’ models are known and widespread, this particular model is quite innovative, as it allows a reduction in waiting time for CYP who are experiencing a crisis, with swift access to appropriate crisis help, flexibility with care, and has improved efficiency and CYP satisfaction [30].
- *The ‘New Beginnings’ crisis recovery model* [21] was created as a recovery model for inpatient settings, with a flexible and recovery-orientated approach. The model is based on the idea that continuous exposure to a persistent problem contributes towards crisis [21]. To resolve CYP crisis, the model utilised interventions to stabilise adolescents by managing their disorganisation, applied systemic functional analysis of presented problems and identified the systemic functional analysis of change required [21]. However, the model is no longer operational due to the reorganisation of the local NHS Trust [21]. The crisis recovery model shows potential to be adapted in community-based settings to manage crisis and reduce the need for in-patient settings [21].
- Additionally, *the Supported Discharge Service* (SDS) is a mixed model between intensive and assertive community treatment that shows a promising reduction in the need for hospitalisation or emergency admission, and self-harm rates and improved school reintegration in comparison to care as usual [27]. The use of such community models may help in reducing a need for hospital or A&E admissions. This particular model may be used as an alternative to the inpatient setting with a degree of caution if applied to other treatment models [27].
- The last model identified in this systematic review represents a complementary and non-clinical model that may act as an alternative to both in-patient setting and crisis services [29]. *The UK Club House model* of mental health recovery is a community mental health service model that supports YP with complex mental health needs to reintegrate them back into society [29, 42]. Pardi and Willis [29] found that in some cases, the use of clubhouses can be a suitable alternative to acute and emergency settings. Even though the model is utilised in non-clinical settings, the clubhouse model signposts individuals to appropriate mental health services where appropriate. Moreover, the flexible and fluid approach of the model aids early intervention and prevention of CYP in crisis. In addition, this particular model could bridge the gap in transition of CYP between CAMHS and AMHS services [29]. However, there is a clear need to investigate the fidelity of the clubhouse model further [42].

#### CYP satisfaction with alternative models

In terms of satisfaction, the One Stop Shop model [30] highlighted increased service user satisfaction and positive service experiences, as well as accessibility and flexibility in comparison to the previous service provision. Similar favourable CYP satisfaction with service provision is visible in the case of the Recovery model [21], while in the case of the SDS, the CYP satisfaction did not differ in comparison to treatment as usual [27]. The CYP satisfaction data were not reported for the York model [26], while in the case of the Clubhouse model, the YP expressed more positive experiences in comparison to experiences with other mental health services they received [29].

#### Interventions applied to a mental health crisis

Three interventions applied to urgent and emergency care from the USA [31–33] and one from the UK [22] were identified.

The Family-Based Crisis Intervention (FBCI) [33] was initially developed for Emergency Departments (ED) to prevent unnecessary hospital admission, and provide patients and their families’ stabilisation intervention followed by signposting and treatment in the community-based setting, thereby avoiding hospital admission [33].

Similarly, the SAFETY program [31] is the brief CBT family intervention, devised for ED’s for treating suicide attempt in YP. The phase 1 of the study reported support for the safety, feasibility, and benefits of the SAFETY intervention, with statistically significant improvements on measures of hopelessness, suicidal behaviour, depression, and youth social adjustment in the intervention group [31]. However, further evaluation of the intervention efficacy and effectiveness is needed.

In contrast, resilient therapy (RT) [22] presents an outcome-focused approach toward developing and improving the resilience of CYP and their families. The RT is designed to improve children’s functioning, and it is also a reflexive tool that can be applied in many different contexts [22]. The main advantage of the RT lies in an adapted language, which is easily understood by CYP, i.e. the use of magic, potions, spells and remedies.

Lastly, the clinical measure of emotional distress dispositions is assessing youth crisis events in both residential and community settings using the Child and Adolescent Needs and Strengths (CANS) intervention-oriented instrument [32]. The finding from this study indicated emotional distress disposition could be clinically measured, and can be a valuable tool for assessing and early detecting CYP behavioural disruption in both residential and community setting [32].

#### CYP/parental satisfaction with identified interventions

Limited satisfaction and improvement in outcomes are reported only in two studies [31, 33]. The SAFETY intervention highlighted that both CYP and their parents reported high satisfaction rates associated with their treatment [31]. Conversely, the FBCI stated that patient and parents reported an improvement in individual and family functioning, and gratitude for being treated by the FBCI [33].

#### Tele Mental Health (TMH)—telepsychiatry and mobile application solution applied to urgent and emergency care

Four studies looked into TMH applications that are being applied to urgent and emergency care. A Canadian study [39] indicated that telepsychiatry is both reliable and cost-effective method for assessment and follow up in the geographically remote areas. Similarly, an American study [35] indicated that the use of telepsychiatry shows clinical and operational efficiency in ED’s by demonstrating that TMH improved access to speciality healthcare services, and increased system capacity, while promoting the delivery of appropriate care in remote and rural areas [35].

In contrast, there is potential in the RCT study protocol [41], which aims to investigate a self-management application for CYP who are experiencing a mental health crisis (suicide ideation). Similar technological endeavour has been noted in the Australian study [38], which created in the cooperation with YP foundations for the first eMental Health clinic.

#### Satisfaction with TMH

The US and Canadian study reported high satisfaction with the use of telepsychiatry [35, 39]. Telepsychiatry is perceived as CYP friendly with a high degree of CYP/Parental acceptability and improved service experience [39]. Similar high outcomes with regards to parental and staff satisfaction with acceptability, effectiveness and efficiency of TMH were reported in the US study [35].

#### Parental and CYP experiences of accessing mental health crisis services

Three studies [25, 36, 37] were focused predominantly on the parents, carers and relatives of individuals who underwent mental health crisis, while two studies [28, 34] were focusing solely on the CYP experiences of undergoing crisis care. The last study was exploring the experiences of both CYP and parents [40]. The analysis resulted in 68 analytical themes, from which we derived five related domains: barriers, emotions and emotional reactions, experiences, needs and what appropriate crisis service should be. The predominant overarching themes between parental and carers and CYP experiences were identified and summarised in “Appendix” (Table 4).

#### Summary of qualitative findings

##### Barriers

Eight barriers were perceived and experienced by parents, while two barriers were experienced by CYP that prevented successful engagement with mental health crisis services and positive mental health outcomes. For CYP, a combination between stigma and fear of opening up is identified as a barrier that can prevent engagement or even create disengagement from further contact with the service [34] (Tables 2, 3).

**Table 2:** What appropriate crisis service should be according to views from parents and CYP

Theme	Parents CYP
Appropriate crisis service should be:	Community-based with a strong relationship between the hospital and the community to provide services In an ideal world, there would not be any such thing as different mental health agencies, it would just be one cohesive thing, and maybe there would be different locations A need for greater flexibility emerged as a key finding along with the concept of immediate real-time services as a necessary shift from the traditional medical mode Authentic youth/caregiver engagement and delivery of services through a flexible, real-time system of care that emphasises prevention and recovery-oriented community-based services Solution: Adaptive recovery-oriented and real-time system of care that integrates hospital and community sectors

**Table 3:** Thematic analysis (domains and themes)

Theme	Parents/carers/relatives	Children and young people
Barriers	Lack of communication from providers Inadequate support from crisis services Fear of confidentiality breaches Lack of involvement with care planning Concerns over the inconsistency of crisis services establishing whether their children are in crisis or not Perception not being listened to Concerns that their parental experiences and observations are not taken into account Reluctance to become involved with help-seeking	Fear of opening up with crisis services Stigma about seeking help from crisis services
Emotions and emotional reactions	Powerlessness Exclusion Frustration Great anxiety Worry Sense of isolation Suffering Complex feelings of guilt and loyalty Feeling abandoned	Powerlessness Exclusion Frustration Anxiety Worry Fear of opening up The feeling of not knowing
Experiences	Lack of understanding The sense of being lost Not being listened or understood Felt often tossed between the crisis assessment services Lack of choice Traumatic and Terrifying experiences The sense of battling through the overall experience Experience of rejection Being told that a child hasn’t relapsed Frequent changes of staff members ‘Double deprivation’ by not receiving appropriate support Being told child not in crisis	Lack of understanding The sense of being lost Not being listened or understood Felt often tossed between the crisis assessment services Lack of choice Struggle to get appropriate help or any help from crisis Disengagement Being Judged Being honest perceived as damning Difficult experiences Lack of therapeutic alliance with crisis staff, Short appointments seen as negative experiences Frequent changes in the staff members Telling their problem more than once (story) In crisis and out of control
Needs	Need to be respected and listened by crisis providers Need to be more assertive Need to battle through the crisis services Need to be signposted to appropriate parental help or support network Need for development of a coping mechanism for dealing with both CYP crisis and mental health crisis services	Need to be respected and listened by crisis providers A need to be treated as a human being A need for safe expression of feelings Need for crisis providers to show that they care

In contrast, a larger number of barriers are evident for parents, carers or relatives of CYP who are being treated by mental health crisis services. For example, a lack of communication from the mental health crisis service providers is a theme that was evident throughout all three studies and is also one of the main reasons for parental dissatisfaction [36, 37]. This ties in with other subthemes such as a lack of involvement with care planning; a perception of not being listened to and not taking into account parental experiences and observations. Parents and carers in two studies reported that they felt they received inadequate support from the mental health crisis provider [25, 36].

##### Emotions and emotional reactions

Findings from this particular domain revealed the complex, and often identical emotional reactions that are reported both by parents and CYP. For example, the sense of frustration, powerlessness, worry, anxiety are often results of the barriers to access and uncertainty which results from the lack of information and appropriate engagement with service provider [34]. Furthermore, parents reported experiences of high burden as a consequence of dealing with a CYP who are undergoing a mental health crisis and crisis service itself at the same time. High level of carers burden was often associated with a sense of isolation, suffering, and feelings of being abandoned by the crisis provider while travelling through the crisis care system [40].

##### Experiences

Both positive and negative experiences with crisis provision were a theme expressed in all six articles. Moreover, a lack of understanding or choice, coupled with the sense of being lost in the system, a consequence of being thrown between different crisis assessment services and not being listened or understood are themes that commonly expressed by both parents and CYP. Furthermore, often staff changes are reported both in CYP and parental experiences, which consequently created an impact on the therapeutic alliance, as well as a need to tell their story on multiple occasions [40].

Additionally, parents and carers often characterised their experiences as terrifying or traumatic [25], while being rejected by the crisis services on several occasions due to staff perceptions that their child is not in crisis or not experiencing relapse [25, 37]. The best way to summarise the parental experiences would be to describe their journey through the crisis services as ‘battling through the system’ [37]. Similar experiences were shared by CYP, who characterised their experiences as difficult, ‘in crisis and out of control’, struggle to get any help from the crisis services, and being judged by the staff members [28, 40]. Besides, short appointments were often seen as a negative experience while being honest was perceived as damning [34, 40]. All these factors led some CYP to experience disengagement from the crisis service [34, 40].

##### Needs

The range of different needs were identified for both CYP and parents such as a need to be listened to and respected by the care provider [34, 37, 40]. Furthermore, parents expressed a set of different needs that parent must have to survive the journey through crisis service. Need for development of a coping mechanism for dealing with both CYP crisis and mental health crisis services, as well as need to become more assertive is reported [37].

Additionally, parents did express that their child’s crisis has a negative impact not just on the parents, but also on the whole family [36]. Therefore, there is a need to be signposted by the crisis service to appropriate parental or family support network [36]. In the case of the CYP, they expressed the need for safe expression of their feelings, being taken seriously, treated as human beings and being showed that crisis staff do care for them [28, 34, 40].

##### CYP and parental perception of what appropriate crisis service should be

Both parents and CYP expressed a positive experience of being treated in the community setting [34, 36]. CYP and Parental opinions were that mental health services should be all encompassed under one roof, with excellent links between hospital and community, with different hubs across the community, using a flexible (non-traditional medical model) approach that emphasises early prevention and recovery [40].

## Discussion

In total, 19 studies were identified in this review. We divided these into four domains: alternative models, interventions applied to mental health crisis, telepsychiatry and mobile applications for urgent and emergency mental health help, and CYP and parental satisfaction and experiences of accessing urgent and emergency mental health services. A surprisingly small number of studies (*n* = 5) focused on new alternatives to inpatient settings or urgent and emergency care models. Additionally, studies that explore the accessibility, acceptability and satisfaction of the CYP and their families with alternatives models are scarce. However, the utilisation of mobile and internet technologies to improve access to mental health services for CYP is increasing, as evidenced by more studies in recent years. Lastly, some of the interventions identified have the potential to be utilised in mental health crisis treatment and may help to reduce hospital admissions and pressure on A&E departments.

We found evidence that synthesised models may be suitable alternatives to inpatients settings. Specifically, we identified two community-based models [26, 30] that offered promising alternatives to hospital-based settings for treating CYP. These are organised in line with the recommendations from the Future in Mind [43] and Five Year Forward view for mental health [44] policies, which state that service providers should be responsive, community-based, and provide improved access with a single point of entry in addition to 24/7 urgent and emergency provision for CYP in crisis. The main innovations of these two models lie predominantly in their accessible, multi-disciplinary triage approaches, their partnerships with both statutory and voluntary sectors and their fully integrated services which enable smooth navigation through the care pathways for CYP [26]. Additionally, the recovery model and support discharge service are also synthesised models that offer a unique approach whereby CYP are treated in community-based settings on the basis of an individual’s needs.

The need for such community-based models is supported with parental and CYP experiential findings that were synthesised as part of this review, which highlighted CYP preferences of being treated in community-based services rather than in hospital or clinical-based settings [40]. These particular findings are in line with previously conducted systematic reviews that emphasise the need for providing mental health treatment in the least restrictive environment [12, 13, 15]. Additionally, intensive community models of service provision promise an alternative to inpatient care for CYP who are affected with mental health issues [12, 13, 15].

Surprisingly, Club house models, despite being non-clinical, perform better in reducing CYP hospitalisation than some clinical models. This is in line with the findings another recently published review, which highlights the potential of the Club house models to decrease re-admission of YP to hospital settings [42]. However, with the evidence currently available, Club house models may be considered more as a complementary model rather than alternatives to both in-patient and crisis services. However, Club house models may have the potential to reduce the reliance of CYP on crisis services and improve the experiences of YP transitioning from CAMHS to AMHS [29]. Nevertheless, further research is required to evaluate the fidelity of the Clubhouse models with appropriate methodological approaches. This is also supported by another recently published review [42].

In the case of four identified models, there is an evident degree of satisfaction of CYP with newly developed services as well as better treatment outcomes. This also corresponds with the findings from Kwok et al. review [12], which clearly stated that more positive CYP experiences could contribute towards higher engagement with providers and better outcomes for both CYP and their parents. Similarly, in the case of the Club house model, the YP indicated high satisfaction with the model, primarily due to not being judged and their opinions and contributions being valued [29].

However, the reported satisfaction with the alternative models as mentioned above does not provide a full understanding of their accessibility and acceptability of those models. This corresponds with the findings from Sheppard et al. [13], which reported similar issues in their systematic reviews, such as a lack of qualitative research that investigated the acceptability of alternative models to inpatient settings.

Furthermore, it is clear from the results of this systematic review, that there is a need for further research with regards to what constitutes appropriate interventions and treatment for CYP experiencing a mental health crisis. Parental qualitative experience and satisfaction indicate that their children are often perceived as not in crisis or not suitable for crisis admission by services, despite being in the crisis or experiencing a relapse [25, 36, 37]. Parental reports also highlight concerns over conflicting diagnosis between different clinicians and the inability of some staff to recognise the crisis [25, 36]. Therefore, there is an evident need for a clear definition of what defines mental health crisis and what particular criteria CYP needs to satisfy to be classified as in crisis [37].

Identified and synthesised interventions in this systematic review showed that most interventions could be applied to urgent and emergency mental health care with CYP. For example, both the Family-based crisis intervention and the SAFETY program are short-term in duration of treatment and such can be successfully delivered both in A&E and outpatient community settings and, therefore, reduce the need for hospitalisation and inpatient admission. Furthermore, these two interventions decrease the carer’s burden, while showing improvement in functioning and increased satisfaction by both CYP and their familes. When the whole family receives support and intervention during a crisis event, there is a visible improvement with levels of satisfaction with service provision, a reduction in both burden and stress in carers, empowerment of family members and improved communication and overall functioning [25, 31, 33, 45].

Separate to the specific interventions, new TMH approaches have been identified. First, the Telepsychiatry models are well established and widely used, especially in the remote and rural areas [46], and may help towards reducing pressure to A&E’s and hospital admissions, by providing timely access to mental health provision. However, several previous reviews have highlighted that there is limited evidence of the effectiveness and efficiency of telepsychiatry or computer-based treatment applications, despite their promising potentials [47–49]. Nevertheless, there is evidence that telepsychiatry is feasible, acceptable and well tolerable for the CYP population [49]. However, telepsychiatry treatments according to some authors should not be used as a sole treatment option; instead, it should complement other mental health models [50]. Second, there are an increasing number of new web and mobile applications that have the potential for use in urgent and emergency mental health services, while some may offer alternatives to inpatient settings, such as Myplan and eMental health. Utilising such technology could offer many potential benefits, such as improved access, reduced waiting times and improved quality of mental health provision for CYP. This is in line with a recent meta-analysis, which clearly supported mobile health interventions for CYP, stating that these interventions seem viable [51].

Finally, the qualitative data provide some understanding of CYP and parental experiences with access and satisfaction with urgent and emergency mental health care which is consistent with findings from a previous systematic review conducted by Shepard et al. [13].

At present, it is clear that some barriers exist that prevents access to mental health crisis provision, and contribute towards disengagement from existing crisis care [52]. From the qualitative data, it is visible that a lack of crisis support coupled with a lack of communication may increase a sense of burden and may result in a lack of confidence in the mental health service providers, which may lead towards disengagement from the service or a reluctance to become involved with any other service [36, 37].

Taking into account the emotional responses from both CYP and their parents, it is clear that some mental health crises can produce unpleasant and traumatic experiences. However, if parents and CYP are taken seriously, fully supported during their mental health crisis treatment journey, and if their experience of mental health crisis treatment is improved, this may contribute towards a reduction of negative experiences or emotional reactions. Often, changes of the staff members can be a cause of concern as this may have a considerable effect on the therapeutic alliance. The importance of the therapeutic alliance is well documented and supported with research evidence, which shows that a good therapeutic alliance is the strong predictor of the positive treatment outcomes [53]. Changes of staff members can contribute towards the need for CYP to repeat their story, and become disengaged from future care. Moreover, Future in Mind recommends that CYP should tell their story only once [43].

## Strengths and limitations

The main strength of this review is the synthesis of experiences and satisfaction of CYP and their families, which, according to our knowledge is the first attempt of reporting the accessibility, acceptability and satisfaction with alternative models to inpatient settings, and urgent and emergency care. Furthermore, we applied a search strategy that resulted in consistent numbers of identified articles in several additional searches. Adherence to the PRISM [24] standards allowed us to maintain methodological rigour. Additionally, the authors employed AMSTAR [54, 55] to check the reliability, validity and methodological quality of this systematic review.

During the process of screening articles, the authors noticed numerous articles in Dutch, German, Swedish and Norwegian that we could not assess, since our protocol criteria required only publications in English to be taken into account, which could be considered a limitation.

Additionally, the qualitative studies included utilised small sample sizes and therefore it may be difficult to extrapolate from their findings for the wider population. Moreover, identified parental experiences and satisfaction related predominantly to females and mothers, with few data from males and fathers.

## Implication for future research

This review found a relative lack of both CYP and parental/carer experiential data in the existing literature. This lack of experiential data is particularly evident in the case of males and fathers, which should be investigated further. It is clear that more research is required on the accessibility, acceptability and satisfaction of service users with alternatives to inpatient settings, and urgent and emergency care. There are also grounds for future research into the TMH applications to mental health crisis and this area appears to be promising and developing rapidly. Lastly, further research could be conducted into the fidelity of Club house models to establish whether they could serve as an alternative or complementary model to clinical models of urgent and emergency care. Additionally, future research could also try to investigate whether Club house models could help improve transition experiences of CYP from CAMHS to AMHS services.

## Conclusion

In this review, we identified 19 studies that we divided into 4 domains: alternative models; CYP/parental satisfaction and experiences of accessing urgent and emergency mental health services; interventions applied to mental health crisis and telepsychiatry/mobile applications for urgent and emergency mental health. Our findings showed that alternative models to inpatient or acute settings may be feasible alternatives for some CYP. We found that CYP had increased satisfaction with alternative models in comparison with care as usual. This was in agreement with previously conducted systematic reviews. However, parental experiential data identified high levels of parental burden and a range of complex emotional reactions associated with engagement with crisis services. Importantly, both parental and CYP experiences highlighted a number of perceived barriers associated with help-seeking from crisis services. Furthermore, the identified and synthesised interventions in this systematic review showed that most interventions could be applied to urgent and emergency mental health care with CYP. However, it is clear from the results of this systematic review, that there is a need for further research to understand what constitutes appropriate interventions and treatment for CYP experiencing a mental health crisis. Moreover, there is limited evidence of the effectiveness of TMH interventions, despite them being widely used. However, recent evidence shows that TMH interventions may be viable, feasible, acceptable and well tolerable for CYP populations. Lastly, this review showed that there is a lack of research evidence investigating the accessibility, acceptability, effectiveness and satisfaction of CYP and their parents with alternative models of mental health crisis provision.

### Electronic supplementary material

Below is the link to the electronic supplementary material.

Supplementary material 1 (DOCX 25 kb)

Supplementary material 2 (XLSX 32 kb)

## Appendix

See Table 4.

**Table 4:** Searching strategy (Medline-Ovid)

#	Searches
1	(child or adolescen$ or youth$ or teenage$ or “young people”).mp. [mp = title, abstract, original title, name of substance word, subject heading word, floating sub-heading word, keyword heading word, organism supplementary concept word, protocol supplementary concept word, rare disease supplementary concept word, unique identifier, synonyms]
2	mental health crisis.ti,ab.
3	mental health crises.ti,ab.
4	(mental health emergency or mental health emergencies).ti,ab.
5	(psychiatric adj (crisis or crises or emergenc* or acute or intensive)).ti,ab.
6	(mental$ adj disorder$).ti,ab.
7	(mental$ adj ill$).ti,ab.
8	(psychiatric adj (crisis or crises or emergenc* or acute or intensive)).mp. [mp = title, abstract, original title, name of substance word, subject heading word, floating sub-heading word, keyword heading word, organism supplementary concept word, protocol supplementary concept word, rare disease supplementary concept word, unique identifier, synonyms]
9	psychopathology.mp.
10	or/2–9
11	ambulatory care.mp.
12	residential treatment.mp.
13	home care service$.mp.
14	psychiatric hospital*.mp.
15	community mental health service*.mp.
16	inpatient*.mp.
17	community service*.mp.
18	wraparound.mp.
19	psychotherapy.mp.
20	early intervention.mp.
21	crisis intervention.mp.
22	foster home care.mp.
23	continuity of patient care.mp.
24	(alternative adj (inpatient or in-patient)).mp.
25	assertive community treatment*.mp.
26	mobile mental health crisis.mp.
27	(multi-systemic or multisystemic).mp. [mp = title, abstract, original title, name of substance word, subject heading word, floating sub-heading word, keyword heading word, organism supplementary concept word, protocol supplementary concept word, rare disease supplementary concept word, unique identifier, synonyms]
28	virtual mental health.mp.
29	respite center.mp.
30	respite service.mp.
31	outpatient treatment.mp.
32	child$ mental health service$.mp.
33	mental health treatment*.mp.
34	mental health hospital admission.mp.
35	mental health treatment outcome*.mp.
36	or/11–35
37	user experience.mp.
38	subjective experience.mp.
39	patient satisfaction.mp.
40	patient perspective.mp.
41	or/37–40
42	1 and 10 and 36 and 41
43	limit 42 to (english language and humans and yr = ”1990–Current”)
