# Supplementary material for: Experiences and satisfaction of children, young people and their parents with alternative mental health models to inpatient settings: a systematic review
Source: Eur Child Adolesc Psychiatry. 2019 Oct 21;29(12):1621–33. doi: 10.1007/s00787-019-01420-7 (PMC7641947; doi:10.1007/s00787-019-01420-7)
Supplement: Supplementary file 1 — Supplementary material 1 (DOCX 25 kb) [file 787_2019_1420_MOESM1_ESM.docx]

## Included

## Screening

Records identified through database searching
(n = 477)

Additional records identified through other sources
(n = 23)

## Identification

Records after duplicates removed
(n = 495)

Records screened
(n = 495)

Records excluded
(n = 235)

Different Language: 8

Adult population (main sample): 29

Treatment of physical health: 19

Satisfaction with either medical model or treatment (Physical health): 25

Mental health Inpatient setting: 32

Primary care (focus on physical health): 17

Not about alternatives to inpatient care, urgent or emergency mental health provision and treatment: 42

Only abstracts available: 7

Systematic Reviews: 6

Not about CYP mental health: 45

Book Chapter: 1

Conference abstracts: 4

Full-text articles assessed for eligibility
(n =260)

## Eligibility

Full-text articles excluded, with reasons
(n =241)

No mental health or crisis: 51

Wrong Population: 83

Inpatient setting: 19

Less than 50% of CYP in the sample: 13

Wrong outcomes: 75

Studies included in qualitative synthesis
(n = 19)

Alternative models: 5

Alternative/Crisis intervention: 4

CYP/Parents/Carers experiences: 6

Telepsychiatry/ Crisis mobile apps: 4
